# Gamma-Glutamyltransferase Level and Risk of Hypertension: A Systematic Review and Meta-Analysis

**DOI:** 10.1371/journal.pone.0048878

**Published:** 2012-11-07

**Authors:** Cun-Fei Liu, Yu-Ting Gu, Hai-Ya Wang, Ning-Yuan Fang

**Affiliations:** Department of Geriatrics, Renji Hospital, Shanghai Jiao Tong University School of Medicine, Shanghai, China; Cardiff University, United Kingdom

## Abstract

**Background:**

Several prospective observational studies suggest that gamma-glutamyltransferase(GGT) level is positively associated with risk of hypertension. However, these studies draw inconsistent conclusions. Therefore, we conducted a systematic review and meta-analysis to evaluate the exact association between GGT level and subsequent development of hypertension.

**Methods:**

We searched Pubmed, Embase, and Science Citation Index (ISI Web of Science) for prospective cohort studies examining the association between GGT level and hypertension. Then, pooled effect estimates (RRs) for the association between GGT level and hypertension were calculated.

**Results:**

A total of 13 prospective cohort studies including 43314 participants and 5280 cases of hypertension were included. The pooled RR of hypertension was 1.94(95%CI: 1.55–2.43; P<0.001) when comparing the risk of hypertension between the highest versus lowest category of GGT levels. Moreover, the risk of hypertension increased by 23% (summary RR: 1.23; 95%CI: 1.13–1.32; P<0.001) per 1 SD logGGT increment. Subgroup analyses showed significant positive associations in each subgroup except in ≧160/95 subgroup (RR: 2.56, 95%CI: 0.87–7.54; P = 0.088) and nondrinkers subgroup (RR: 1.76, 95%CI: 0.88–3.53; P = 0.113). Sensitivity analyses showed no single study significantly affects the pooled RRs. No publication bias was found in our meta-analysis.

**Conclusions:**

GGT level is positively associated with the development of hypertension. Further studies are needed to confirm our findings and elucidate the exact mechanisms between GGT level and the incidence of hypertension.

## Introduction

Gamma-glutamyltransferase(GGT) is a common biomarker of liver injury and alcohol consumption [1]. However, recent epidemiologic and clinical studies have also found a close association between GGT level and risk of cardiovascular disease, diabetes, and metabolic syndrome [2], [3], [4]. The relationships between them were further strengthened by published meta-analyses [5], [6], [7].

In the past twenty years, the association between GGT and risk of hypertension has been deeply investigated in cross-sectional and longitudinal studies [8]–[20]. However, these studies drew inconsistent, even opposite conclusions. For example, Kim et al [17] found a significant association between GGT quartiles and hypertension only in the drinkers and non-overweight subgroups, while Stranges et al [15] showed that higher GGT level increased the risk of hypertension in both noncurrent and current drinkers. Because of these inconsistent findings, we therefore conducted a systematic review and meta-analysis to attempt to solve the problem.

## Methods

### Search Strategy

The Pubmed, Embase, and Science Citation Index (ISI Web of Science) databases were searched to collect all publications on the association between GGT and hypertension (last search update: 15th June 2012) without language restriction. The following search terms were used: (Gamma-glutamyltransferase[MeSH] OR Gamma-glutamyltransferase[All Fields] OR gamma-GT[All Fields] OR GGT[All Fields] OR GGTP[All Fields]) AND (Hypertension[MeSH] OR Hypertension [All Fields] OR “Essential Hypertension”[All Fields] OR “Primary Hypertension”[All Fields] OR “High blood pressure”[All Fields]. Similar search terms were applied to Embase and the Science Citation Index. Moreover, reference lists were further retrieved from published articles to avoid missing any relevant studies. Our meta-analysis was performed according to the Meta-Analysis of Observational Studies in Epidemiology (MOOSE) guidelines [21].

### Study selection

Studies were included if they satisfied the following criteria: 1) the study design should be a prospective design; 2) GGT level in baseline was reported and hypertension as the outcome of interest; 3) the relative risk(RR) or odds ratio(OR) with 95% confidence intervals(CIs) were provided or obtained by calculation.

### Data extraction

Two authors extracted information independently and disagreements were resolved by discussion and consensus. The following data were extracted from each included article: name of the first author, year of publication, country of origin, mean age of the populations, gender component, numbers of participants and cases, durations of follow-up, diagnostic criteria for ascertainment of hypertension, most fully adjusted effect estimates from multivariable for the highest versus the lowest group of GGT level with corresponding 95%CI and study-specific adjusted confounding factors. When the effect estimates of the same population were reported in different follow-up durations, we only included the data with the longest follow-up time.

### Statistical analysis

Relative risk (RR) and 95% CI were chosen as the effect estimate to assess the association between GGT and hypertension. We calculated the combined RR and 95%CI using the most-adjusted RRs(by comparing the highest versus lowest category of GGT level). Heterogeneity among studies was examined by using chi-square-based Q test and I^2^ test. When significant heterogeneity (P value<0.05 and I^2^>50%) was detected, the pooled RR and 95%CI would be estimated in a random-effect model. Otherwise, a fixed-effect model was chosen. Subgroup analyses were further carried out by race/ethnicity, sex, sample sizes, durations of follow-up, definition of hypertension and number of adjusted confounding factors. In addition, we also analyzed combined RR per 1 SD (standard deviation) logGGT increment in three studies which presented their results (RRs) in per 1 SD logGGT increment.

A sensitivity analysis was performed to evaluate whether the results were markedly affected by a single study. Publication bias was evaluated with Begg's and Egger's test, with a P value >0.05 considered as no significant. All statistical analyses were conducted with Stata software, version 11.0 (Stata Corp, College Station, Texas, USA). A P value<0.05 was considered statistically significant.

## Results

### Literature search

**Figure 1:**
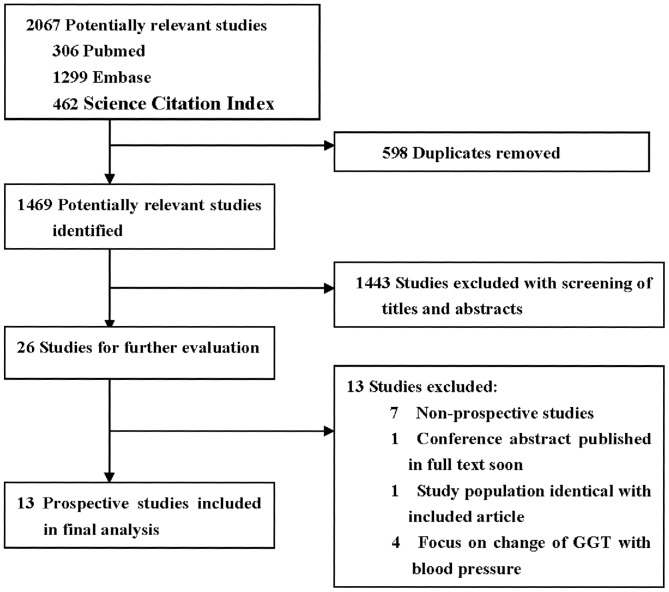
Flow diagram of included studies of meta-analysis.

The flow diagram of literature search was shown in Figure 1. 1469 potentially relevant articles were initially identified. Of the 1469 articles identified, 1443 papers were excluded based on titles and abstracts. The remaining 26 articles were then further evaluated by reviewing full text. 13 studies were excluded due to the following reasons: 7 studies not a prospective design, 2 studies repeated with included studies, and 4 studies focused on the change of GGT over time with blood pressure. Finally, 13 articles [8]–[20] were included in our meta-analysis.

### Study characteristics

Thirteen prospective cohort studies involving 43314 participants and 5280 cases of hypertension were included in our meta-analysis. The characteristics of the 13 articles were summarized in Table 1. Among 13 studies, 10 studies were conducted in Asia, 2 in North-America, and 1 in Europe. The durations of follow-up ranged from 3 to 15 years. As to diagnosis of hypertension, hypertension was defined as blood pressure ≧160/95 mmHg, 140/90 mmHg, 130/85 mmHg and/or taking antihypertensive medications. Twelve studies including 15 data points (as 3 studies provided their results for men and women separately) offered the RRs and 95%CIs of highest versus lowest category of GGT level. Moreover, three studies [12], [16], [20] reporting their results in per 1 SD logGGT increment were exclusively analyzed.

**Table 1:** Characteristics of studies included in meta-analysis.

Study	Country	Case/Total [age(y)]	Gender M/F	Definition of Hypertension	Follow-up (years)	Comparison (Highest vs Lowest, U/L)	Adjusted RR(95%CI)	Adjustment for covariates
Yamada [11]; 1991	Japan	29/1393 (35–54)	1393/0	≧160/95	5	≧50 vs <50	4.52(2.17–9.40)	None
Miura [20], 1994	Japan	36/77 (30–69)	77/0	≧140/90, meds	10	≧20 vs <10	5.82(1.83–18.56)	Age, SBP, DBP, alcohol consumption.
						Per 1 SD logGGT increment	1.41(1.09–1.83)	
Lee [14], 2002	Korea	169/8170 (25–50)	8170/0	≧160/95, meds	4	≧50 vs ≦9	1.5(0.8–2.8)	Age, BMI, smoking, drinking, exercise, family history of hypertension, SBP or DBP, the change of BMI, drinking during four years.
Lee [13], 2003	USA	708/4704 (18–30)	NA	≧140/90, meds	15	>36 vs ≦12	1.5(1.0–2.2)	Age, sex, race, study center, BMI, alcohol consumption, cigarette smoking, physical activity, systolic blood pressure, insulin.
Stranges [15], 2005	USA	195/897 (39–79)	587/310	≧140/90, meds	6	(39–55) vs ≦14	2.1(1.1–4.0)	Age, gender, race, average of alcohol, smoking status, BMI, physical activity, systolic blood pressure.
Ander [10], 2007	France	492/2273 (NA)	1129/1144	≧130/85, meds	3	M: ≧49.4 vs <19.7; F: ≧23 vs <12.6	M:1.76(1.06–2.92); F:1.38(0.87–2.20)	Age
Jo [9], 2009	Korea	2170/17281 (NA)	11659/5622	≧130/85, meds	4	M:>38 vs <19; F:>15 vs <9	M:2.44(2.07–2.88); F:1.49(1.14–1.94)	Age
Jimba [18], 2009	Japan	288/1027 (49±8)	NA	≧130/85, meds	3	≧42 vs ≦24	1.15(0.76–1.73)	Age, sex, alcohol habit, BMI
Hwang [19], 2010	Korea	83/293 (54.1±8.9)	115/176	≧140/90, meds	5	M: >46 vs <17; F: >19 vs <9	M:0.5(0.1–2.8); F:7.8((2.4–25.0)	Age, education, BMI, alcohol intake, cigarette smoking, exercise, salt intake, family history of hypertension, ALT
Cheung [16], 2011	China	126/708 (47.3±9.7)	428/280	≧140/90, meds	5.3	M: ≧31 vs ≦20; F: ≧20 vs ≦13	2.68(1.36–5.26)	Age, sex, systolic blood pressure at baseline, follow-up duration, BMI, triglycerides, HDL cholesterol, HOMA-IR, CRP, fibrinogen, current smoking, change in BMI
						Per 1 SD logGGT increment	1.38(1.05–1.81)	
Xu [8], 2011	China	119/285 (NA)	NA	≧130/85, meds	3.5	(41–68) vs <16	1.55(0.72–3.31)	Age, sex
Onat [12], 2012	Turkey	476/1423 (33–84)	735/678	≧140/90, meds	4	Per 1 SD logGGT increment	1.20(1.10–1.31)	Age, sex, menopause, BMI, alcohol usage
Kim [17], 2012	Korea	389/4783 (44±5.8)	3246/1537	≧140/90, meds	3	(29–51) vs ≦12.9	2.638(1.259–5.528)	Age, sex, alcohol amount, smoking status, physical activity, BMI, baseline glucose, uric acid, HDL, LDL, TG, Hs-CRP, baseline systolic blood pressure

Abbreviations: NA: not applicable; M, Man; F, Female; BMI, body mass index; ALT, alanine aminotransferase; HOMA-IR, homeostasis model assessment index of insulin resistance; CRP, C-reactive protein; HDL, high-density lipoprotein cholesterol; LDL, low-density lipoprotein cholesterol; TG, triglyceride.

### Meta-analysis

**Figure 2:**
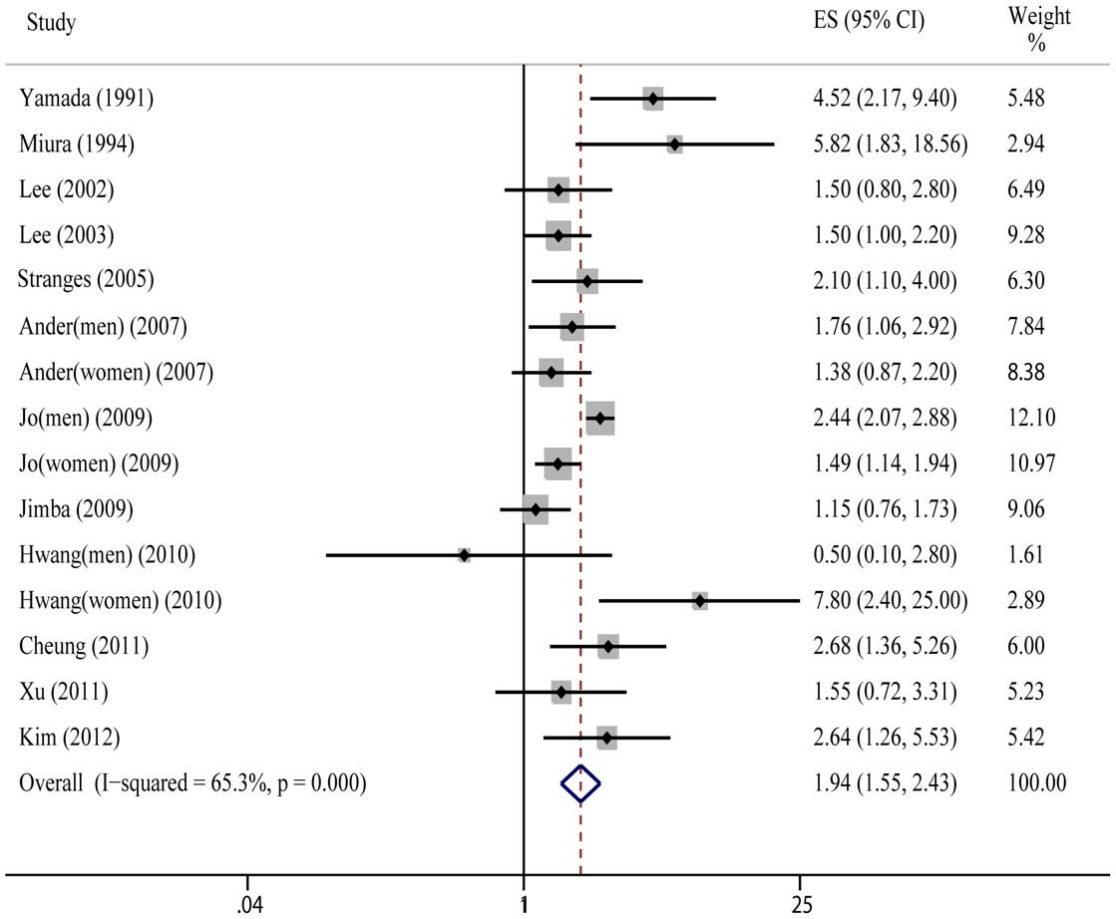
Meta-analysis of risk of hypertension between highest vs lowest category of GGT.

As is shown in Figure 2, the pooled RR of hypertension was 1.94(95%CI: 1.55–2.43; P<0.001) for the highest versus lowest category of GGT level; statistical heterogeneity was found in the study results (Q = 40.29, P = 0.000, I^2^ = 65.3%).

As above, three studies [12], [16], [20] provided their results in per 1 SD logGGT increment. Overall, risk of hypertension increased by 23% (summary RR: 1.23; 95%CI: 1.13–1.32; P<0.001) per 1 SD logGGT increments without evident heterogeneity (Q = 1.82, P = 0.403, I^2^ = 0%).

### Subgroup and sensitivity analyses

The effects of GGT level on risk of hypertension in different subgroups are shown in Table 2. Overall, the positive association between GGT level and risk of hypertension was consistently observed in each subgroup except in ≧160/95 subgroup (RR: 2.56, 95%CI: 0.87–7.54; P = 0.088) and nondrinkers (RR: 1.76, 95%CI: 0.88–3.53; P = 0.113).

**Table 2:** Subgroup analyses of GGT level and risk of hypertension.

Group	Number of studies	RR(95%CI)	P value for effect estimates	P value for heterogeneity	I^2^(%)
All studies	15a	1.94(1.55–2.44)	<0.001	<0.001	65.3
Race/ethnicity					
Asians	11	2.14(1.58–2.90)	<0.001	<0.001	71.6
Non-Asians	4	1.59(1.25–2.03)	<0.001	0.727	0
Gender					
Men only	6	2.29(1.56–3.37)	<0.001	0.033	58.8
Women only	3	1.91(1.06–3.43)	0.031	0.022	73.8
Both men and women	6	1.70(1.28–2.24)	<0.001	0.203	31.0
Drinking status					
Nondrinkers	5	1.76(0.88–3.53)	0.113	0.136	42.8
Drinkers	4	2.39(1.29–4.44)	0.006	0.051	61.4
Durations, y					
≤5	11	1.87(1.42–2.45)	<0.001	<0.001	70.8
>5	4	2.23(1.40–3.53)	0.001	0.110	50.2
Sample size					
≤1000	5	2.86(1.48–5.54)	0.002	0.053	57.2
>1000	10	1.77(1.39–2.24)	<0.001	0.001	67.8
Definition of HBP					
≧160/95	2	2.56(0.87–7.54)	0.088	0.025	80.1
≧140/90,	7	2.44(1.54–3.86)	<0.001	0.026	58.1
>130/85	6	1.63(1.21–2.18)	0.001	0.001	74.9
Adjustment for covariates					
≤5 factors	8	1.90(1.40–2.57)	<0.001	<0.001	75.3
>5 factors	7	2.05(1.40–3.00)	<0.001	0.066	49.2

a: twelve studies including 15 data points.

**Figure 3:**
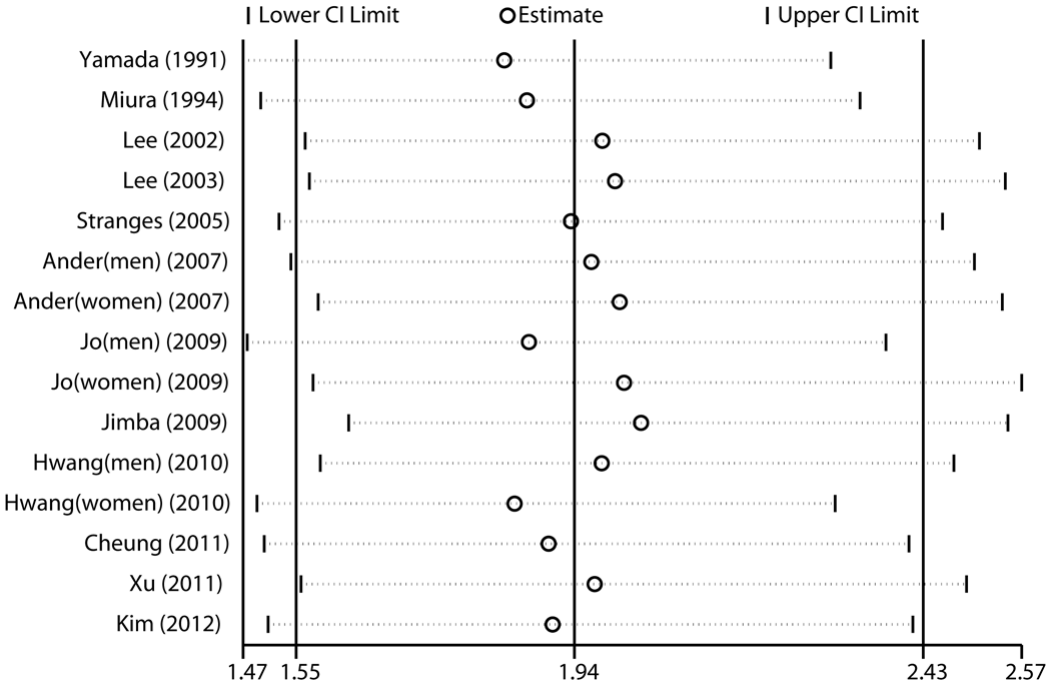
Sensitivity analyses results of given named study omitted.

To evaluate the impact of single studies on the combined results, we conducted sensitivity analyses by omitting one study at a time and reassessed the summary RR for the remaining studies. As is shown in Figure 3, no single study significantly affects the pooled RRs

### Publication bias

**Figure 4:**
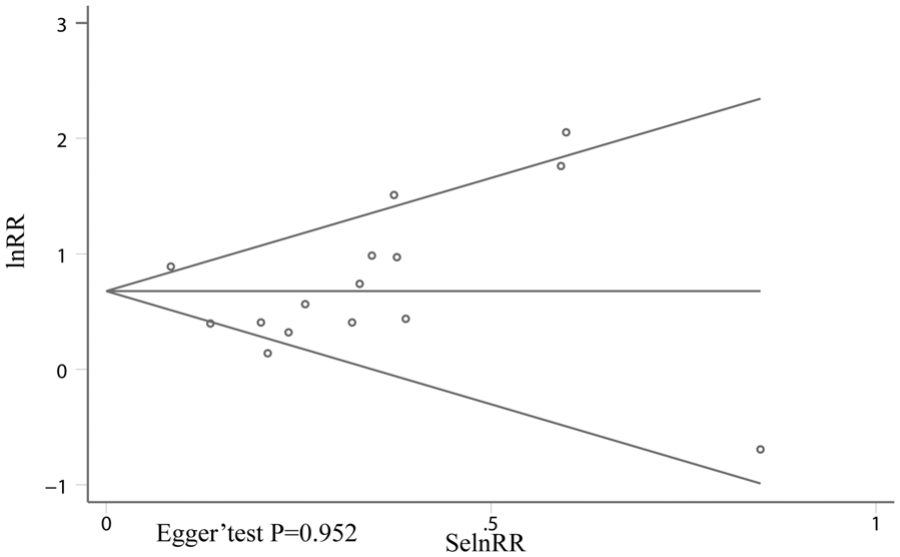
Begg' funnel plot analysis of publication bias.

Begg's and Egger's test were performed to test if there was publication bias. As is shown in Figure 4, no publication bias was found (Egger' test: P = 0.952)

## Discussion

Recent studies suggested that GGT was a novel biomarker of cardiovascular risk. As to the relationship between GGT and hypertension, previous studies have drawn inconsistent conclusions. In this study, we comprehensively evaluated the exact association between GGT level and risk of hypertension. Our present systematic review and meta-analysis of 13 prospective studies showed a significant positive association between GGT level and risk of hypertension (RR: 1.94, 95%CI: 1.55–2.43). Moreover, our results also showed that risk of hypertension increased by 23% (95%CI: 1.13–1.32) per 1 SD logGGT increment. Subgroup analyses showed that the positive association between GGT level and risk of hypertension consistently existed in each subgroup except in ≧160/95 subgroup (RR: 2.56, 95%CI: 0.87–7.54; P = 0.088) and nondrinkers subgroup (RR: 1.76, 95%CI: 0.88–3.53; P = 0.113).

In our subgroup analyses, an interesting phenomenon was observed: the positive association between GGT level and risk of hypertension was more significant in Asians and male subgroups than that in non-Asians and female subgroups. As is commonly known, Asians have more prevalence of hepatic diseases such as hepatitis B or C, while men are more prone to drink alcoholic beverages. Accordingly, subgroup analysis in nondrinkers showed an increased trend but did not reach a statistical significance (RR: 1.76, 95%CI: 0.88–3.53; P = 0.113). Therefore, although most studies in our meta-analysis have adjusted alcohol habits or liver diseases, the possibility that the positive association of GGT and risk of hypertension is explained by alcohol consumption or liver diseases is not completely excluded. However,three studies [15], [17], [19] performed within normal range of GGT levels showed that relative risk is still much higher in highest category of normal range of GGT levels than that in lowest category (RR: 2.44, 95%CI: 1.15–5.20; P = 0.021). Hence, further prospective studies with much more strict inclusion criteria should be conducted to evaluate a more accurate relationship between GGT level and the development of hypertension.

Exact mechanisms that link GGT with hypertension are not fully elucidated, however, several possible explanations are as follows: Longitudinal study showed that GGT is positively associated with inflammation markers such as fibrinogen, C–reactive protein and F2-isoprostanes [13]. In addition, a study found that GGT was expressed in human atherosclerotic lesions colocalizing with ox-LDL and foam cells, which could contribute to the progression of atherosclerosis [22]. Lastly, GGT plays an important role in the generation of free radical species through its interaction with iron [23]. Base on the above findings, elevated GGT level could be a marker of inflammation condition and oxidative stress, which were important features of hypertension.

Several limitations in our study should be pointed out. First, although most included studies have adjusted for a series of potential confounders, the possibility of residual or unknown confounding still can not be completely excluded. Second, several studies included in our meta-analysis did not specifically exclude subjects with hepatic diseases or alcohol abuse at baseline, this may confuse the true association of GGT with hypertension as liver damage and alcohol intake could affect GGT levels. However, studies that investigated only in nondrinkers [16] or participants without hepatitis [17] and within normal range of GGT levels [15], [17], [19] also found a positive association between GGT level and risk of hypertension. Third, moderate heterogeneity was observed in our meta-analysis, which is not surprising because of methodological variations, including study population, different ranges of exposure, and number of adjustment factors among studies. To find out source of heterogeneity, we conducted a meta-regression analysis. Regrettably, we did not detect the source of heterogeneity (data not shown). Finally, publication bias may existed for studies with null results as these tend not to be published. However, we did not find evidence of publication bias in our meta-analysis.

In conclusion, our study suggests that GGT level is independently associated with the development of hypertension. However, further studies are needed to confirm our findings and elucidate the exact mechanisms between GGT and the incidence of hypertension.

## References

[1] Whitfield JB (2001) Gamma glutamyl transferase. Crit Rev Clin Lab Sci 38: 263–355. 11563810 10.1080/20014091084227

[2] Nakanishi N, Suzuki K, Tatara K (2004) Serum (gamma)-glutamyltransferase and risk of metabolic syndrome and type 2 diabetes in middle-aged Japanese men. Diabetes Care 27: 1427–1432. 15161799 10.2337/diacare.27.6.1427

[3] Lee DS, Evans JC, Robins SJ, Wilson PW, Albano I, et al (2007) Gamma glutamyl transferase and metabolic syndrome, cardiovascular disease, and mortality risk: the Framingham Heart Study. Arterioscler Thromb Vasc Biol 27: 127–133. 17095717 10.1161/01.ATV.0000251993.20372.40

[4] Ryu S, Chang Y, Woo HY, Yoo SH, Choi NK, et al (2011) Longitudinal increase in (gamma)-glutamyltransferase within the reference interval predicts metabolic syndrome in middle-aged Korean men. Metabolism: Clinical and Experimental 59: 683–689. 10.1016/j.metabol.2009.08.024 19922966

[5] Liu CF, Zhou WN, Fang NY (2012) Gamma-glutamyltransferase levels and risk of metabolic syndrome: a meta-analysis of prospective cohort studies. Int J Clin Pract 66: 692–698. 22698421 10.1111/j.1742-1241.2012.02959.x

[6] Fraser A, Harris R, Sattar N, Ebrahim S, Smith GD, et al (2007) Gamma-glutamyltransferase is associated with incident vascular events independently of alcohol intake: analysis of the British Women's Heart and Health Study and Meta-Analysis. Arterioscler Thromb Vasc Biol 27: 2729–2735. 17932318 10.1161/ATVBAHA.107.152298

[7] Fraser A, Harris R, Sattar N, Ebrahim S, Davey SG, et al (2009) Alanine aminotransferase, gamma-glutamyltransferase, and incident diabetes: the British Women's Heart and Health Study and meta-analysis. Diabetes Care 32: 741–750. 19131466 10.2337/dc08-1870 PMC2660465

[8] Xu Y, Bi YF, Xu M, Huang Y, Lu WY, et al (2011) Cross-sectional and longitudinal association of serum alanine aminotransaminase and (gamma)-glutamyltransferase with metabolic syndrome in middle-aged and elderly Chinese people. Journal of Diabetes 3: 38–47. 21199427 10.1111/j.1753-0407.2010.00111.x

[9] Jo SK, Lee WY, Rhee EJ, Won JC, Jung CH, et al (2009) Serum (gamma)-glutamyl transferase activity predicts future development of metabolic syndrome defined by 2 different criteria. Clinica Chimica Acta 403: 234–240. 10.1016/j.cca.2009.03.035 19332046

[10] Andre P, Balkau B, Vol S, Charles MA, Eschwege E (2007) Gamma-glutamyltransferase activity and development of the metabolic syndrome (International Diabetes Federation Definition) in middle-aged men and women: Data from the Epidemiological Study on the Insulin Resistance Syndrome (DESIR) cohort. Diabetes Care 30: 2355–2361. 17586745 10.2337/dc07-0440

[11] Yamada Y, Ishizaki M, Kido T, Honda R, Tsuritani I, et al (1991) Alcohol, high blood pressure, and serum gamma-glutamyl transpeptidase level. Hypertension 18: 819–826. 1683858 10.1161/01.hyp.18.6.819

[12] Onat A, Can G, Ornek E, Cicek G, Ayhan E, et al (2012) Serum gamma-glutamyltransferase: independent predictor of risk of diabetes, hypertension, metabolic syndrome, and coronary disease. Obesity (Silver Spring) 20: 842–848. 21633402 10.1038/oby.2011.136

[13] Lee DH, Jacobs DR Jr, Gross M, Kiefe CI, Roseman J, et al (2003) Gamma-glutamyltransferase is a predictor of incident diabetes and hypertension: the Coronary Artery Risk Development in Young Adults (CARDIA) Study. Clin Chem 49: 1358–1366. 12881453 10.1373/49.8.1358

[14] Lee DH, Ha MH, Kim JR, Gross M, Jacobs DR Jr (2002) Gamma-glutamyltransferase, alcohol, and blood pressure. A four year follow-up study. Ann Epidemiol 12: 90–96. 11880216 10.1016/s1047-2797(01)00252-6

[15] Stranges S, Trevisan M, Dorn JM, Dmochowski J, Donahue RP (2005) Body fat distribution, liver enzymes, and risk of hypertension: evidence from the Western New York Study. Hypertension 46: 1186–1193. 16203871 10.1161/01.HYP.0000185688.81320.4d PMC1694276

[16] Cheung BM, Ong KL, Tso AW, Cherny SS, Sham PC, et al (2011) Gamma-glutamyl transferase level predicts the development of hypertension in Hong Kong Chinese. Clin Chim Acta 412: 1326–1331. 21466796 10.1016/j.cca.2011.03.030

[17] Kim NH, Huh JK, Kim BJ, Kim MW, Kim BS, et al (2012) Serum Gamma-Glutamyl Transferase Level Is an Independent Predictor of Incident Hypertension in Korean Adults. Clin Exp Hypertens in press. 10.3109/10641963.2012.665539 22471622

[18] Jimba S, Nakagami T, Oya J, Wasada T, Endo Y, et al (2009) Increase in gamma-glutamyltransferase level and development of established cardiovascular risk factors and diabetes in Japanese adults. Metab Syndr Relat Disord 7: 411–418. 19419267 10.1089/met.2008.0082

[19] Hwang JH, Shin JY, Chun B, Lee DH, Kim KY, et al (2010) Association between gamma-glutamyltransferase and hypertension incidence in rural prehypertensive adults. J Prev Med Public Health 43: 18–25. 20185979 10.3961/jpmph.2010.43.1.18

[20] Miura K, Nakagawa H, Nakamura H, Tabata M, Nagase H, et al (1994) Serum gamma-glutamyl transferase level in predicting hypertension among male drinkers. Journal of human hypertension 8: 445–449. 7916380

[21] Stroup DF, Berlin JA, Morton SC, Olkin I, Williamson GD, et al (2000) Meta-analysis of observational studies in epidemiology: a proposal for reporting. Meta-analysis Of Observational Studies in Epidemiology (MOOSE) group. JAMA 283: 2008–2012. 10789670 10.1001/jama.283.15.2008

[22] Franzini M, Corti A, Martinelli B, Del CA, Emdin M, et al (2009) Gamma-glutamyltransferase activity in human atherosclerotic plaques–biochemical similarities with the circulating enzyme. Atherosclerosis 202: 119–127. 18486136 10.1016/j.atherosclerosis.2008.03.023

[23] Lee DH, Blomhoff R, Jacobs DR Jr (2004) Is serum gamma glutamyltransferase a marker of oxidative stress? Free Radic Res 38: 535–539. 15346644 10.1080/10715760410001694026

