# Correction: Gamma-Glutamyltransferase Level and Risk of Hypertension: A Systematic Review and Meta-Analysis

**DOI:** 10.1371/annotation/8015aa06-f752-4a7e-ba31-4fcc1ea50b6c

**Published:** 2013-05-10

**Authors:** Cun-Fei Liu, Yu-Ting Gu, Hai-Ya Wang, Ning-Yuan Fang

There was an error in the Funding statement.

The correct Funding statement is: The work was supported by the National Natural Science Foundation of China (30971264). The funders had no role in study design, data collection, analysis, decision to publish or preparation of the manuscript. 

